# Bridging the phenotypic and genetic data useful for integrated breeding through a data annotation using the Crop Ontology developed by the crop communities of practice

**DOI:** 10.3389/fphys.2012.00326

**Published:** 2012-08-25

**Authors:** Rosemary Shrestha, Luca Matteis, Milko Skofic, Arllet Portugal, Graham McLaren, Glenn Hyman, Elizabeth Arnaud

**Affiliations:** ^1^Genetic Resources Program, Centro Internacional de Mejoramiento de Maiz y TrigoTexcoco, Edo. de México, Mexico; ^2^Bioversity InternationalMaccarese, Rome, Italy; ^3^Generation Challenge Programme, Centro Internacional de Mejoramiento de Maiz y TrigoTexcoco, Edo. de México, Mexico; ^4^Centro Internacional de Agricultura TropicalCali, Colombia

**Keywords:** Crop Ontology, breeding trait, plant phenotype, trait dictionaries, breeding fieldbook, data annotation, integrated breeding platform, crop community of practice

## Abstract

The Crop Ontology (CO) of the Generation Challenge Program (GCP) (http://cropontology.org/) is developed for the Integrated Breeding Platform (IBP) (http://www.integratedbreeding.net/) by several centers of The Consultative Group on International Agricultural Research (CGIAR): bioversity, CIMMYT, CIP, ICRISAT, IITA, and IRRI. Integrated breeding necessitates that breeders access genotypic and phenotypic data related to a given trait. The CO provides validated trait names used by the crop communities of practice (CoP) for harmonizing the annotation of phenotypic and genotypic data and thus supporting data accessibility and discovery through web queries. The trait information is completed by the description of the measurement methods and scales, and images. The trait dictionaries used to produce the Integrated Breeding (IB) fieldbooks are synchronized with the CO terms for an automatic annotation of the phenotypic data measured in the field. The IB fieldbook provides breeders with direct access to the CO to get additional descriptive information on the traits. Ontologies and trait dictionaries are online for cassava, chickpea, common bean, groundnut, maize, Musa, potato, rice, sorghum, and wheat. Online curation and annotation tools facilitate (http://cropontology.org) direct maintenance of the trait information and production of trait dictionaries by the crop communities. An important feature is the cross referencing of CO terms with the Crop database trait ID and with their synonyms in Plant Ontology (PO) and Trait Ontology (TO). Web links between cross referenced terms in CO provide online access to data annotated with similar ontological terms, particularly the genetic data in Gramene (University of Cornell) or the evaluation and climatic data in the Global Repository of evaluation trials of the Climate Change, Agriculture and Food Security programme (CCAFS). Cross-referencing and annotation will be further applied in the IBP.

## Introduction

In recent years, sequence information has become readily available for a variety of crop species. However, a gap is emerging between the physical genome information and the quantitative information regarding phenotypes. It is becoming clear that the application of quantitative genetic information by researchers and breeders is limited by a lack of standard nomenclature used to describe both crop development and agronomic traits. Without either a nomenclature or information, which provides the equivalence links between trait descriptions, it is hard to compare information from Quantitative Trait Loci (QTL) and association studies in a way that permits systematic transfer of knowledge about genotype-phenotype relationships among crops or between crops.

In the case of crop breeding programs, plant breeders repeatedly measure a large number of traits in order to understand the crop phenotype, based on variation in genotype and environment. Some traits are common across crops whereas some other traits are crop specific such as anthesis silking interval (ASI) for maize. Common traits across crops can be measured with different methods and scales. Likewise, one trait could be measured under several environmental conditions at different growth stages within a crop. Therefore, the management of crop characterization and evaluation data in databases at the global level is always complex and critical. The situation is more complex for traits like resistance to disease or to abiotic stresses such as drought and salinity tolerance. For example a plant pathologist could score stem rust disease in the greenhouse at seedling stage or in the field (adult plants for severity and incidence) by artificial inoculation of pathogen or via natural infestation using different scoring rating scales. To enable comparison of these different types of measurements related to a single trait, and to support future modeling of the correlation among several traits the following are required: (1) that a nomenclature and controlled vocabularies in the form of ontologies are applied in databases and knowledge bases and (2) the data generated by the trials/experiments are properly annotated by crop communities practiced in using validated trait names, and adjusted to the recommended methods of measurement and scales. Data annotation is the addition of metadata (i.e., ontological terms) that describe the data file and possibly the data point. Phenotype and genotype data annotation enable researchers to attach information and data to a botanical term, a development stage and a trait name. It can also be used to specify the process through which trait data has been obtained and its provenance. Although annotation of genetic data is commonplace, data produced via phenotyping studies are usually not annotated using a controlled vocabulary to facilitate their integration into multi-crop platforms.

## Application of the integrated breeding crop ontology in crop research

**Figure 1:**
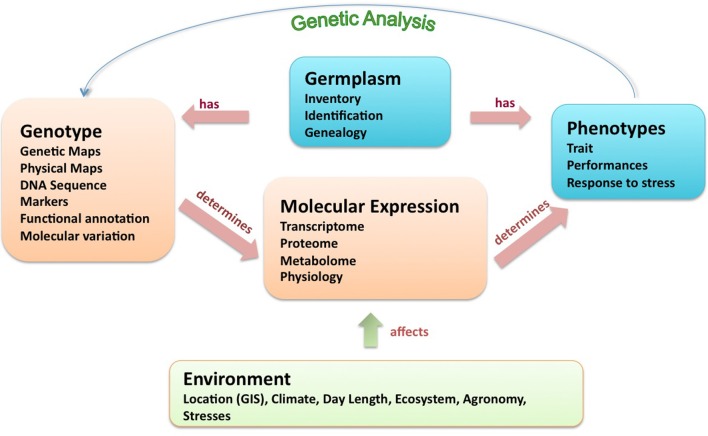
**Biological relationships in germplasm research adapted from Bruskiewich et al. (2006)**.

The fundamental scientific question underlying research on diverse genotypes of any plant species is “What is the causal relationship between genotype and phenotype?” DNA is transcribed into RNA, which is either bioactive itself (as non-coding RNA gene products) or is translated into peptides that form part of protein gene products. Ultimately, these products act as structural elements, genetic regulatory control factors, or modulators of the biochemical fluxes within metabolic and physiological pathways, at the sub-cellular, tissue, organ, and whole organism level. This sum total of molecular expression integrates the overall structural and behavioral features of the plant—its “phenotype.” The unfolding of this story also has an essential environmental context, including biotic (ecosystem) and abiotic (geophysical) factors modulating expression in a variety of ways via diverse sensory and regulatory mechanisms in the plant. Various classes of experimental data associated with this tapestry of germplasm function are summarized in Figure 1.

Phenotypes and genotypes can be characterized at various levels of abstraction and resolution (Bruskiewich et al., 2006). In the case of plant phenotypes, it includes measurements of traits at different growth stages, in various environments and treatment conditions. Genotypes include laboratory measurements of DNA and simple observations of visible phenotypes. The molecular variation measured by genotyping can be neutral or biologically significant. Neutral molecular variation generally involves markers that simply exhibit DNA structural polymorphism that is usefully applied to answer basic questions on the extent of similarity between germplasm samples (i.e., “fingerprinting” experiments) or on the chromosome location of a marker (i.e., “mapping” experiments). Answering such questions will often lead to deeper exploration of germplasm, such as evolutionary studies, practical management of plant crosses, and genetic resource management. Whatever the nature of phenotype and genotype measurements, the primary task is to completely capture and accurately codify the raw and derived phenotype and genotype data. The role of the ontology is precisely to support the description of all the pathways between the gene and the expression of the trait, enabling data interpretation (Shrestha et al., 2011). The Crop Ontology (CO) provides additional terms and descriptions of traits, along with methods and scales that complement the Gene Ontology (GO; http://geneontology.org), Plant Ontology (PO; http://plantontology.org) and Trait Ontology (TO; http://www.gramene.org/) for bridging a wider set of annotated genetic, genomic, and phenotypic data with formalized phenotype descriptions and leading to data discovery. Documentation of protocols related to phenotypic data is very important for enabling comparison across crops, environments and plant growth stages and the CO aims to provide comprehensive information about the trait and the measurement of the trait.

## The crop ontology (CO) and the trait dictionaries in the integrated breeding fieldbook

**Figure 2:**
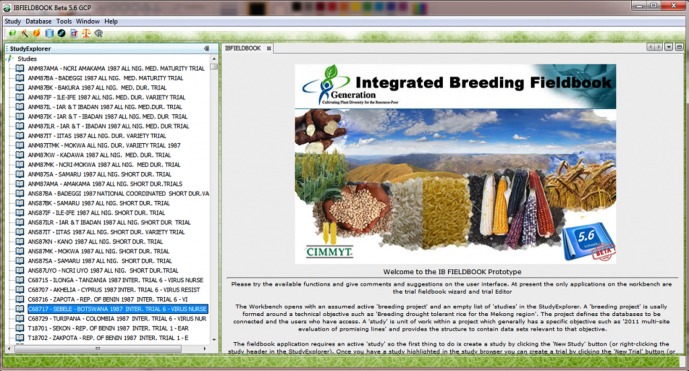
**Integrated Breeding Fieldbook for capturing trait measurement with mobile devices**.

The Integrated Breeding Platform (IBP; http://integratedbreeding.net/) is developed by the Generation Challenge Programme (GCP; http://www.generationcp.org/) for crop breeders. The objective of the IBP is to provide access to modern breeding technologies, breeding material, and related information and services, in a centralized and functional manner. This should improve plant breeding efficiency in developing countries and facilitate the adoption of molecular breeding approaches (Delannay et al., 2011). The Integrated breeding fieldbook (referred to in the text as the IB Fieldbook, Figure 2) supports the harmonized capture of trait measurements in the evaluation sites and their integration in the crop databases. The fieldbook's trait template is based on the trait dictionary and includes a link to the corresponding trait name in the IB CO.

The objectives of the integrated workflow between the IB Fieldbook, the Trait Dictionary and the CO are (1) for breeders and data managers to define a standard list of traits; (2) for breeders to access more information on the trait and the protocols used for measurement when defining their evaluation experiment; (3) to provide an automatic annotation of the data captured by breeders via the CO terms. The CO, in combination with the crop trait dictionaries, provides a tool to foster the phenotypic and genotypic data curation and annotation by the communities of practice (CoP) of several crops using validated common trait names, particularly breeders' traits, protocols, and scales.

### Creating trait dictionaries for the crop databases and the fieldbooks

The IB Fieldbook and the crop databases based on the International Crop Information System (ICIS) contain the trait dictionaries to support the harmonization of the trait measurements across the phenotyping sites and the data annotation across databases. The trait dictionaries and the ontology are embedded into the crop databases for cassava, chickpea, rice, maize, wheat, and soon for banana, groundnut, cowpea, common beans, pigeon pea, and sorghum. Each crop-specific trait ontology and dictionary will be maintained by acrop lead center and/or a crop research community.

To assist breeders an Excel spread sheet template was developed to simplify the process of submitting traits, trait descriptions, allocation of categories or valid ranges and measurement protocols. Utilization of the trait template was very helpful to obtain extended trait information and manage the quality control of trait names within the databases. Multi-location evaluation programs have been conducted in several countries to ensure that trait names are stored in the fieldbooks and databases in several languages. An indicator of the language has also been added to the online trait dictionaries so that crop communities can send trait names in different languages via the basic trait template. The same term identifier will be used for the same trait in different languages, so that different versions of the same trait are referred to as synonyms to facilitate the search of data across languages.

Recently, the trait dictionaries were used to prioritize the traits according to the frequency of use by breeders in their research programs and importance for the crop. The objective was to provide a core standard set of crop specific traits that will appear by default in the crop fieldbook wherever the crop is evaluated. A list of optional traits is also available and can be added by the breeder according to the evaluation objective. All existing trait dictionaries have been uploaded in the CO and are also available for download on each crop page of the IBP website. The harmonization between the CO and the trait dictionaries will be continuously performed by the CoP and the use of the online ontology will be prioritized to avoid deviation from a single reference list of traits, methods and scales.

### Deploying the trait dictionaries annotated with the crop ontology terms

**Figure 3:**
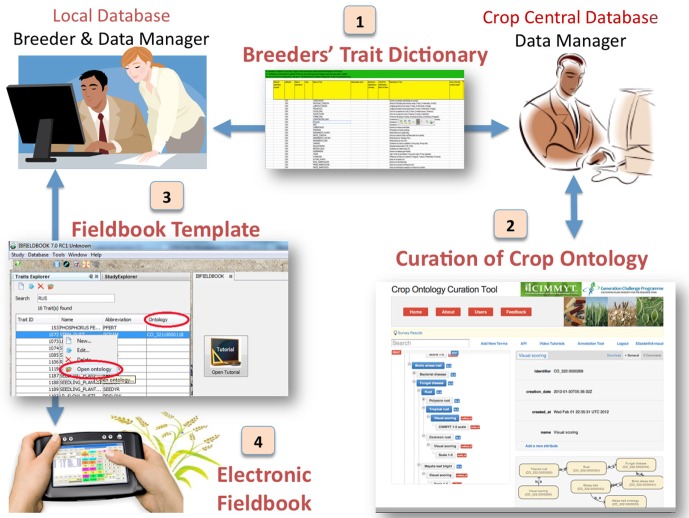
**Trait data flow between the ontology, the crop databases and the field book**.

The schema of the GCP crop database, along with the trait dictionaries, is being deployed within each CoP through the installation of a central database managed by the crop lead center and several local databases installed in the research stations and partners institutions. The trait dictionaries that include the CO terms are embedded into the central database and are maintained by crop data curators. The curator manages the validation and synchronization of trait dictionaries with the online CO curation tool. The local crop databases contain the reference trait dictionaries inherited from the central database that is used to design the field book template for the handheld or the printed form. This data flow (Figure 3) ensures that traits measured in the field are harmonized across sites and are captured within the template format. The CO terms and their identifiers, which are embedded into the fieldbook template, ensure that data are already annotated without any additional effort from the database curator. The annotated data could therefore easily be synchronized from the hand held data capture devise to the local database and then to the central crop database.

## Development of the crop-specific trait ontologies

At present, the CO provides crop-specific trait ontologies for cassava, chickpea, maize, musa, potato, sorghum, rice, wheat, as well as online trait dictionaries for common bean, cowpea, and groundnut developed by the crop lead centers of the GCP challenge initiatives. These simple trait lists built in the form of controlled vocabularies with short descriptions do not fulfill all the requirements for ontology-based access to data. Therefore, the trait dictionaries will be upgraded into ontologies by adding multiple relationships and cross referencing to other major ontologies. Since 2007, the crop-specific ontologies were developed in the crop lead centers, by teams of breeders, biometricians and data managers using the OBO-Edit software promoted by the Open Biomedical Ontology (OBO) communities such as GO (Ashburner and Lewis, 2002; Day-Richter et al., 2007), PO and TO (Jaiswal et al., 2002). By using OBO-Edit, ontology curators are able to construct the ontology from lists of traits, create the necessary multi-relationships between terms, and simultaneously create cross-references with the terms in TO and PO. Multi-relationships between biological terms provide the semantic framework, which is necessary to model the biological pathways, describing the expression of the traits in plants, in various tissues, at different development stages and different environments.

**Figure 4:**
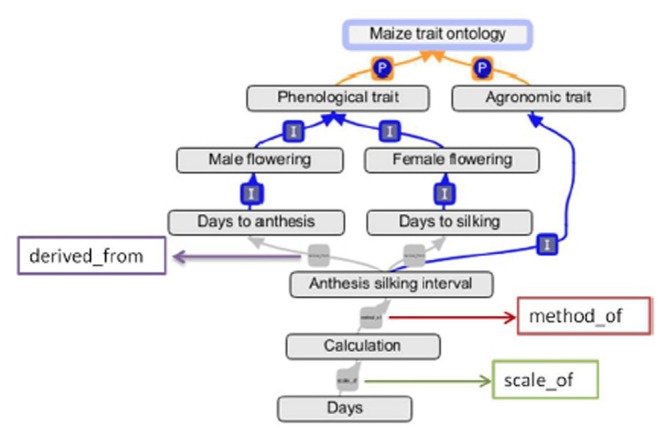
**Representation of the multi-relationships of “Anthesis silking interval” in OBO-Edit**.

The CO describes agronomic, morphological, physiological, quality, and abiotic and biotic stresses related traits of several crops using most common “is_a” and “part_of” relations assigned by OBO-foundry (Shrestha et al., 2010). The methodology, which was applied for developing the PO and TO, was also used for developing the CO. In order to embed methods and scales in the Crop specific ontologies, new ontological relations were created such as “method_ of,” “scale_of,” and “derived_from” for meaningfully describe the traits and their relations to methods and scales (Figure 4).

## The online crop ontology site for a community-based curation and annotation

**Figure 5:**
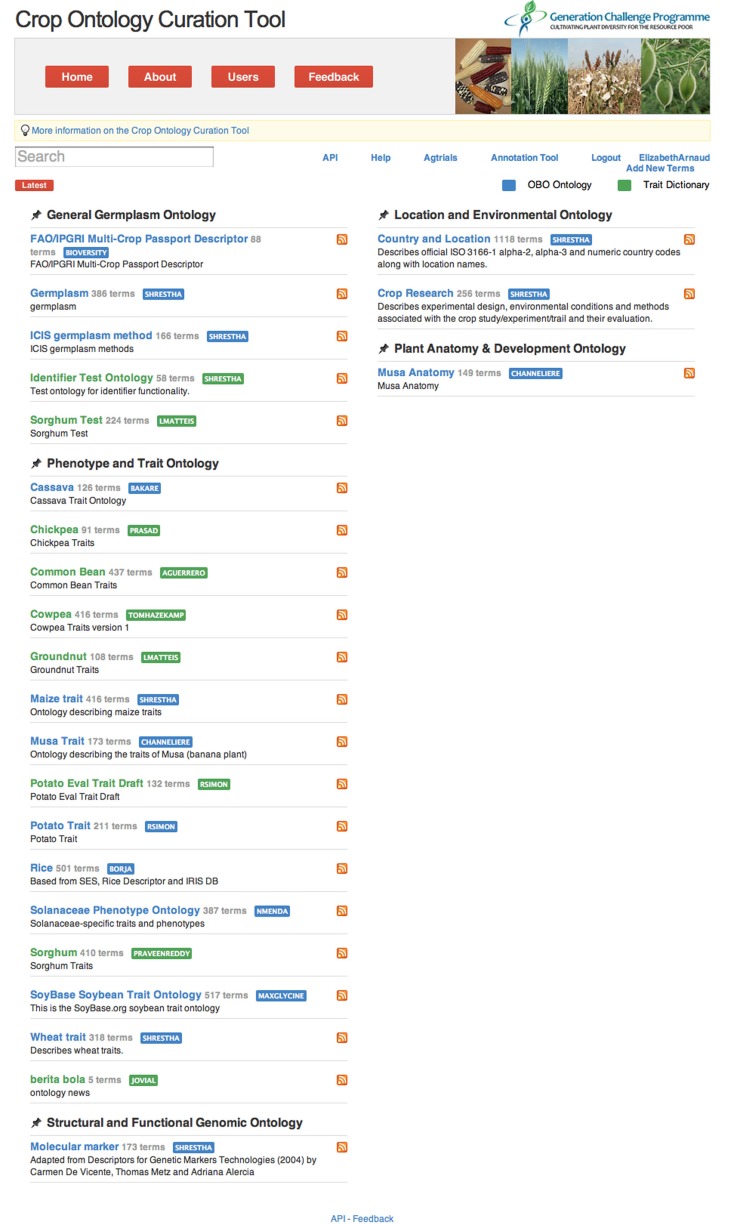
**Crop Ontology homepage (http://www.cropontology.org)**.

In 2011, the new CO website (www.cropontology.org) was released providing a tool for participatory ontology development, curation, and annotation by the crop database curators (Figure 5). Users can browse crop-specific ontologies, access trait definition with the bibliographic reference, synonyms, images, term abbreviation, as well as online cross references to PO, TO and the GCP crop databases. The tool provides features for posting comments and printing trait information. Only crop specific curators are allowed to upload ontologies, add new terms and attributes of traits and edit text to control quality. Video tutorials are available in the website. The code used for the development is hosted on Google App Engine and the versioned code is hosted on GitHub.

**Figure 6:**
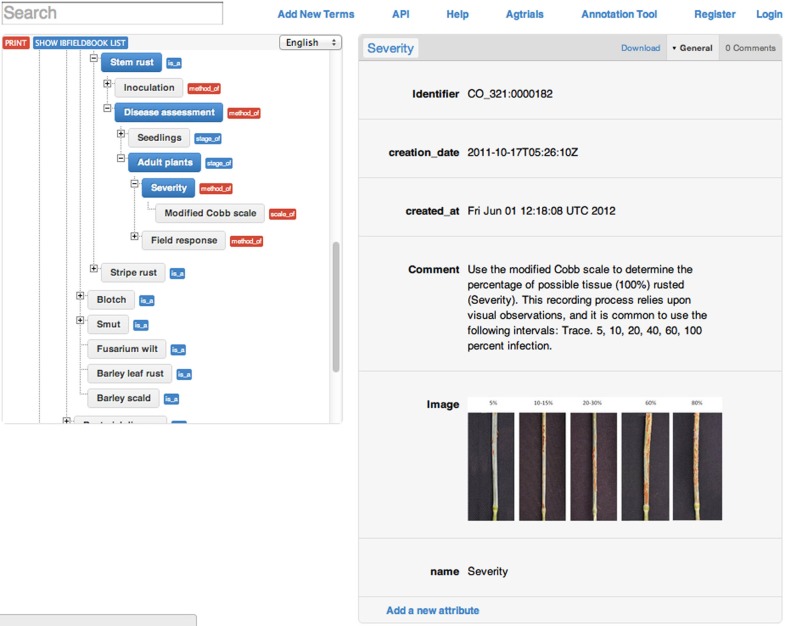
**Online display of new relationships “method_of” and “scale_of” for “stem rust” along with information and images about the scale used for measurement**.

Trait measurement methods are displayed as derived terms of the related trait name with newly created relationship “method_of” and scales are derived terms of their related method with relationship “scale_of” (Figure 6). Providing protocols related to traits facilitates the selection of appropriate terms for data annotation and data exchange across databases.

**Figure 7:**
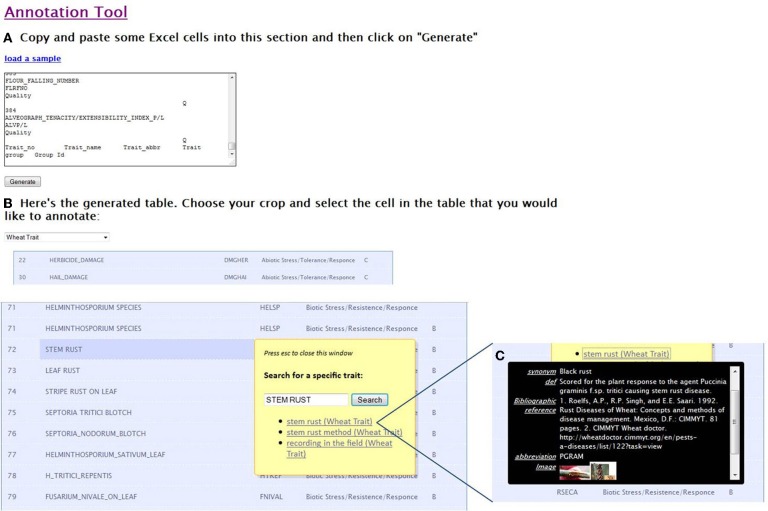
**Screenshot of the online annotation tool showing steps in the annotation process: (A) paste data or metadata to annotate (B) the tool generates a table and user can select one ontology (e.g., maize trait) before annotation (C) information and images about the corresponding ontological term are displayed below the term selected for annotation (e.g., anthesis silking interval). Users can check and validate or reject the proposition**.

The prototype of the online annotation tool was inspired by Terminizer, developed by David Hancock (University of Manchester, http://terminizer.org/). This tool allows the user to associate the ontology terms with existing trait names extracted from the database or text and overcome the heterogeneous manner of naming the traits (Figure 7).

## Expanding the use of the crop ontology into the international community for data discovery

**Figure 8:**
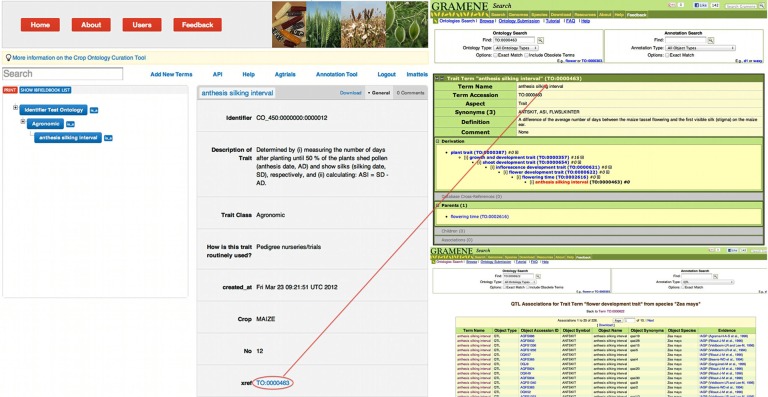
**Direct access to the QTL information associated with the trait “anthesis silking interval” on the Gramene website through the cross referencing link placed in the Crop Ontology**.

New CO terms were submitted for addition to PO and TO. The collaboration will continue through the cross-referencing of PO, TO and CO in order to develop internationally shared crop trait ontology. To extend the access to genetic information, CO curators have cross-referenced most of the traits with synonyms in PO and TO. An important online feature is the active web linkages of these cross-referenced terms that direct users to the corresponding term-specific page on Gramene (Cornell) or on PO and the annotated genetic data (e.g., QTL) associated with the trait (if available) (Figure 8).

The United State Department of Agriculture (USDA) and the Solanaceae Genomics Network (SGN)—who are presently the most interested to cross reference their respective ontology and data with the GCP CO to enable data integration—have uploaded their respective ontology on the online curation tool: the Soybean ontology for Soybase and the Solanaceae ontology.

## An open source server of cross-referenced trait names for data integration

**Figure 9:**
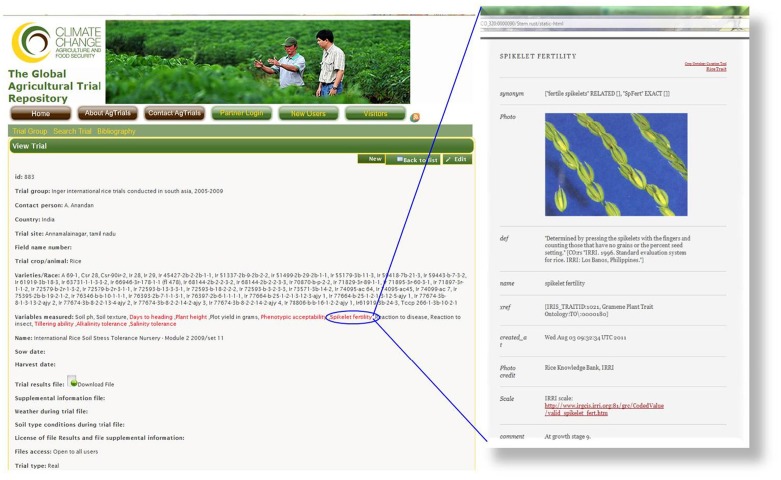
**Screenshot showing the dynamic link from the variable “spikelet fertility” on Agtrials to additional information in the online Rice Ontology**.

The online Integrated Breeding CO is a freely available resource that acts as open-source server for names of traits thanks to an Application Programming Interface (API). The API enables programmatic access to the CO by web sites, web services or data template wizards that can dynamically synchronize their lists of traits with the CO. This synchronization supports the harmonization of data annotation and then enables the discovery of annotated data through web queries based on the ontology terms. The first site to use the API is the Global Agricultural Trial Repository of the CGIAR program on Climate Change for Food and Agriculture Security (CCAFS; http://www.agtrials.org:8080/). The CCAFS initiative dynamically links the names of variables measured during the evaluation of varieties with the CO terms. The objectives are (1) to facilitate the annotation of the data files by users with harmonized trait names; and (2) to provide users with access to detailed information on the variables (Figure 9).

**Figure 10:**
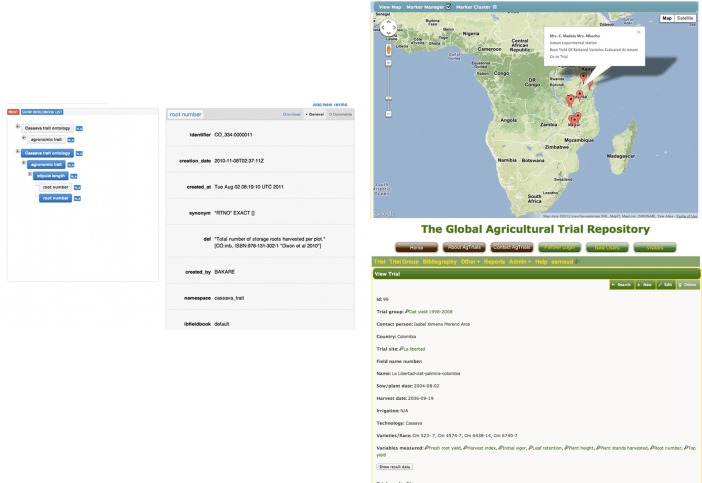
**Mockup of an ontological trait based access to the map of trials on Agtrials**.

This cross-referencing prepares the ground for integration of online data into a single site, and the objective is to integrate this further within the IBP. Integrating the Agtrials website with the CO would provide, for any given trait and crop, access to the phenotypic data combined with geographical and environmental data (Figure 10).

## Conclusions

The development of a GCP CO for breeders' traits is a pioneering activity that was acknowledged by major partners in the agronomic research and in the landscape of phenotype ontology development such as the USDA, the Solanaceae Genomics Consortium, Cornell University, the PO Consortium, the National Center for Biotechnology Information and the NSF Research Coordination Network on Phenotype. The CO development is currently based on Trait dictionaries defined by teams of breeders and data managers for direct use in the IB Fieldbook. This initiative facilitates direct annotation of breeders' data captured in the field and will enable the integration of phenotypic and genetic data sets. It will also help the breeders, when evaluating traits in the field, to access the correct trait information they need, including detailed standard protocols and scales. Thanks to the new online curation and annotation tool, the curators of crop specific ontologies can interactively modify existing trait names or add new ones along with images, methods and scales. A full ontology can easily be uploaded or created online, which encourages partnership for the cross-referencing of terms. Once published online, the cross reference of traits are converted into a web link to directly access related data in other websites like Gramene (University of Cornell) or Agtrials (CCAFS-CIAT). This is the premise of the integration of phenotypic, genotypic and environmental data associated with a given trait. The IBP will further utilize the CO to integrate as much as possible of the genetic data in the genomic data management system with the phenotypic data collected in the GCP phenotyping sites. This online access of the CO provides a useful mechanism for bridging a wider set of annotated genetic, genomic and phenotypic data with formalized phenotype descriptions that will lead to new data discovery.

### Conflict of interest statement

The authors declare that the research was conducted in the absence of any commercial or financial relationships that could be construed as a potential conflict of interest.
